# Increasing dietary soybean trypsin inhibitor protein attenuates nursery pig performance

**DOI:** 10.1093/tas/txaf089

**Published:** 2025-07-11

**Authors:** Kayla A Miller, Joel D Spencer, Hari B Krishnan, Omarh F Mendoza, Michelle N McCallum, Julie A Mahoney, Eric R Burrough, Nicholas K Gabler

**Affiliations:** Iowa State University, Department of Animal Science, Ames, IA 50011, USA; United Animal Health, Sheridan, IN 46069, USA; USDA-ARS, University of Missouri, Columbia, MO 65211, USA; The Maschhoffs LLC., Carlyle, IL 62231, USA; United Animal Health, Sheridan, IN 46069, USA; United Animal Health, Sheridan, IN 46069, USA; Iowa State University, Department of Veterinary Diagnostic and Production Animal Medicine, Ames, IA 50011, USA; Iowa State University, Department of Animal Science, Ames, IA 50011, USA

**Keywords:** nursery pig, soybean, trypsin inhibitor

## Abstract

Trypsin inhibitor proteins are antinutritional compounds innate to soybeans that reduce protein digestibility, amino acid bioavailability, and growth performance of pigs. The objective of this study was to evaluate the impact of increasing levels of dietary trypsin inhibitor unit activity (TIU/mg) on nursery pig growth performance and health. In a 41-d study, 1,140 newly weaned nursery pigs (5.9 ± 0.34 kg BW) were allotted into split sex pens, blocked by body weight, assigned randomly to one of five dietary treatments (n = 19 pens/treatment) varying in TIU/mg concentration, and fed over three dietary phases. Treatments targeted 0.41, 1.32, 2.20, 3.08, and 3.96 TIU/mg of complete feed averaged over the three phases and were achieved by using a corn-soybean meal basal diet with added soybean flour. Analyzed dietary treatments averaged 0.61, 1.22, 2.19, 3.41, and 3.51 TIU/mg. Pen BW and feed disappearance were recorded at the start and end of each phase to calculate ADG, ADFI, and G:F. Fecal consistency was scored and recorded daily. On d 21 of the study, 10 pigs per treatment were sacrificed for intestinal sample collection. Data were analyzed with pen as the experimental unit, the random effect of block, and the fixed effect of TIU, including polynomial contrasts for linear and quadratic effects of 0.61 to 3.51 TIU/mg treatments. No quadratic responses to dietary TIU/mg activity were reported in any parameters. Overall, as active dietary TIU/mg increased, ADG, ADFI, and G:F linearly decreased (*P* < 0.001). Pigs fed the highest level (3.51 TIU/mg) exhibited reduced ADG by 25%, ADFI by 17%, and G:F by 8% compared to pigs fed the lowest level (0.61 TIU/mg). Dietary TIU/mg did not affect fecal consistency, mortality, or removals (*P* > 0.10). Individual and total concentrations of colonic biogenic amines and short chain fatty acids did not differ (*P* > 0.10). Histological lesions of the ileum and colon did not differ (*P* > 0.10). Ileum VH tended to decrease (*P* = 0.078) and CD linearly decreased as TIU/mg increased (*P* = 0.004), but VH:CD and colonic CD were similar (*P *> 0.10). Moderate relationships between TIU intake and G:F (R^2^ = 0.393), caloric efficiency (R^2^ = 0.378), and lysine efficiency (R^2^ = 0.376) were observed. In conclusion, soybean-derived active TIU concentrations negatively impact nursery pig performance above 1.22 TIU/mg, with minimal impacts on intestinal and pig health.

## Teaser

This study aimed to evaluate the effects of active trypsin inhibitor (TIU) protein concentrations on nursery pig performance and health metrics over a 41-d period. Findings from this study indicate that nursery pig performance begins to decline at dietary trypsin inhibitor concentrations exceeding 1.22 TIU/mg of complete feed.

## INTRODUCTION

Trypsin inhibitor proteins are among various antinutritional compounds inherent to legumes, such as soybeans, which serve as a predator defense mechanism for the plant (Guillamon et al., 2008). In soybeans, other known antinutritional compounds include β-conglycinin and glycinin proteins, tannins, lectins, saponins, phytoestrogens, phytates and goitrogens (Liener, 1994). Of particular interest to poultry and pigs are the two primary protease inhibitors, Kunitz trypsin inhibitor (Kunitz, 1946) and Bowman-Birk inhibitor (Bowman, 1944; Birk, 1961). These protease inhibitor proteins impede protein digestion by inhibiting the activity of trypsin and chymotrypsin in monogastric animals (Liener, 1994). This inhibition reduces nutrient bioavailability and attenuates growth rates of animals (Liener, 1976, 1994). Additionally, the reduced bioavailability of amino acids is often linked to an increased incidence of diarrhea in younger pigs due to increased hindgut proteolytic fermentation and luminal osmotic draw (Zhang et al., 2020). The adverse effects of active trypsin inhibitor proteins, including weight loss and nutrient malabsorption, have been documented in rats (Liener, 1953; Gu et al., 2010), chicks (Palacios et al., 2004; Clarke and Wiseman, 2005), and pigs (Combs et al., 1967; Yen et al., 1974; Goebel and Stein, 2011).

Soybean products are predominantly used as protein sources in nursery pig diets. In the United States, soybean meal active trypsin inhibitor unit (**TIU**) concentrations range from 2 to 20 TIU/mg (Kim and Krishnan, 2023). Thus, assuming nursery diets typically contain approximately 15 to 30% soybean meal, TIU concentrations could range from less than 0.5 to 6 TIU/mg of complete feed. Previous studies have examined the impact of varying TIU/mg levels on nursery and grower pig performance and nutrient digestibility. In nursery pigs, Nisley et al. (2024) observed negative effects on growth, feed intake, and feed efficiency above 3 TIU/mg in complete feed, with 5.79 TIU/mg resulting in a 10 kg reduction in body weight over a 42-d growth period compared to the low 0.38 TIU/mg control diet; however, no diarrhea or fecal consistency issues were noted. In grower pigs, Miller et al. (2024) reported linear decreases in performance and nitrogen digestibility with increasing dietary TIU concentrations. Additionally, Royer et al. (2022) found that weight gain, feed intake, and feed efficiency ratio declined at dietary concentrations exceeding 2 TIU/mg. Therefore, the objective of this experiment was to further define the concentration above which active soybean-derived TIU would reduce pig performance, increase body weight variability, and affect the general health of nursery-aged pigs in a commercial setting. It was hypothesized that dietary concentrations above 3 TIU/mg would attenuate nursery pig growth performance and increase body weight variability, fecal looseness, and pig removals.

## MATERIALS AND METHODS

### General

Live animal research was conducted at the United Animal Health Donald E. Orr Jr. Swine Research Farm (Sheridan, IN) from September to October 2024. All procedures were approved by the Iowa State University Institutional Animal Care and Use Committee (IACUC# 23-111) and adhered to the ethical and humane use of animals for research. The facility contained two identical rooms and was temperature regulated and adjusted to the pig’s thermoneutral requirements as the pigs aged. Each room was fully concrete slatted and contained 60 pens (1.40 m × 2.13 m), where each pen provided 0.30 m^2^ per pig, and was equipped with a gate-mounted stainless steel cup waterer and a 3-hole stainless steel feeder (0.61 m × 0.25 m). The facility utilized an automated feed delivery system (Big Dutchman; Big Dutchman North America, Holland, MI).

### Ingredient Analysis and Dietary Treatments

A low TIU soybean meal (ADM Soy Processing, Frankfort, IN) and two soybean flours (White River Soy Processing, Creston, IA) were sourced from commercial soybean processors and utilized in dietary formulations to attain the selected soybean-derived TIU levels. For the soybean flours, one had a target protein dispersibility index (**PDI**) of 20 (20PDI) and the other had a PDI of 70 (70PDI). Soybean flours and soybean meal used for the experiment were analyzed (Table 1) for dry matter (Method 930.15; AOAC, 1999), crude protein (Method 990.03; AOAC, 2002), calcium and phosphorus (Method 985.01 A, B, and D; Method 942.05; AOAC, 2006), neutral detergent fiber (**NDF**, Method 2001.11; AOAC, 2005), acid detergent fiber (**ADF**) based on Goering and Van Soest (1970), and ether extract (Method 2003.05; AOAC, 2006). Dispensable and indispensable amino acids composition of raw soybeans and soybean meal was determined by the Agricultural Experiment Station Chemical Laboratories at the University of Missouri–Columbia (Columbia, MO) by cation-exchange HPLC (L8900 Amino Acid Analyzer, Hitachi High-Technologies Corporation, Tokyo, Japan). Active trypsin inhibitor concentrations (expressed as TIU/mg) of soybean meal, soybean flour 20PDI and 70PDI were determined by methods previously described by Kim and Krishnan (2023). Analyzed values of the 20PDI and 70PDI soybean flours contained 27.6 and 53.3 TIU/mg, 50.83% and 49.49% crude protein, and 2.07% and 2.51% crude fat, respectively, and contained identical amino acid profiles. Further, the soybean meal utilized contained 0.96 TIU/mg.

**Table 1.** Ingredient analysis of soybean meal and soybean flour, as-fed

Parameter	Soybean meal	Soybean flour 20 PDI	Soybean flour 70 PDI
Trypsin inhibitor units, TIU/mg	0.98	24.4	58.7
Dry matter, %	88.2	95.0	94.8
Ash, %	6.3	6.6	6.7
Crude protein, %	46.5	51.3	51.4
Crude fat, %	1.5	2.1	2.5
Acid detergent fiber, %	6.4	3.3	5.3
Neutral detergent fiber, %	7.2	5.7	5.1
Calcium, total, %	0.42	0.30	0.26
Phosphorus, total, %	0.70	0.76	0.68
*Indispensable amino acids, %*			
Arginine	3.41	3.81	3.74
Histidine	1.33	1.43	1.43
Isoleucine	2.35	2.65	2.56
Leucine	3.71	4.06	4.01
Lysine	3.14	3.35	3.39
Methionine	0.64	0.73	0.73
Phenylalanine	2.46	2.71	2.68
Threonine	1.89	1.99	1.97
Tryptophan	0.65	0.55	0.58
Valine	2.44	2.70	2.65
*Dispensable amino acids, %*			
Alanine	2.06	2.28	2.23
Aspartic acid	5.39	5.88	5.87
Cysteine	0.65	0.74	0.78
Glutamic acid	8.67	9.52	9.50
Glycine	2.00	2.20	2.19
Proline	2.35	2.57	2.57
Serine	1.86	2.12	2.13
Taurine	0.14	0.14	0.16
Tyrosine	1.69	1.90	1.92
Total amino acids	46.91	51.44	51.36

Dietary treatments consisted of increasing concentrations of TIU formulated across three dietary phases, where phase 1 (Table 2) was fed from d 0 to 9, phase 2 (Table 3) was fed from d 10 to 22, and phase 3 (Table 4) was fed from d 23 to 41. Phase 1 diet targeted TIU concentrations were 0.70, 1.60, 2.40, 3.20, and 4.00 TIU/mg of complete feed, while Phase 2 and 3 targeted concentrations were 0.26, 1.18, 2.10, 3.02, and 3.94 TIU/mg of complete feed. To achieve these target values, soybean meal and flour were incorporated into a corn-soybean meal-based diet. Dietary treatments were fed in meal form and formulated to be isocaloric and to meet or exceed the nutrient requirements for nursery pigs (NRC, 2012). Additionally, diets were equalized on the total amount of soy products (soybean meal, 20 PDI, and 70 PDI soybean flour) in the diet. Analyzed TIU concentrations were determined for all diets (Table 2, Table 3, and Table 4) as previously described by Kim and Krishnan (2023). Across all three dietary phases, the weighted averages of analyzed dietary TIU based on days per dietary phase were 0.61, 1.22, 2.19, 3.41, and 3.51 TIU/mg of complete feed. All diets were analyzed in duplicates for nitrogen and crude protein content (Table 2, Table 3, and Table 4) using TruMac N (Leco Corporation, St. Joseph, MO, USA).

**Table 2.** Diet composition of phase 1, as fed basis^1^

Ingredient, %	Dietary Treatment (TIU/mg)^*^				
	0.61	1.22	2.19	3.41	3.51
Corn	43.41	43.52	43.59	43.60	43.60
Soybean meal	10.30	6.70	4.10	4.10	4.10
Soybean flour (20PDI)	-	3.60	5.40	2.60	-
Soybean flour (70PDI)	-	-	0.80	3.60	6.20
Basemix^2^	42.50	42.50	42.50	42.50	42.50
Bovine plasma	2.00	2.00	2.00	2.00	2.00
Limestone	0.24	0.24	0.25	0.25	0.25
Monocalcium phosphate	0.21	0.18	0.16	0.16	0.16
Salt	0.32	0.32	0.31	0.31	0.31
Vitamin/Trace mineral premix^2^	0.02	0.02	0.02	0.02	0.02
L-Lysine	0.26	0.24	0.22	0.22	0.22
DL-Methionine	0.08	0.07	0.06	0.06	0.06
L-Threonine	0.06	0.05	0.05	0.05	0.05
L-Tryptophan	0.07	0.07	0.07	0.07	0.07
L-Valine	0.03	0.01	-	-	-
L-Isoleucine	0.09	0.07	0.06	0.06	0.06
Zinc oxide	0.42	0.42	0.42	0.42	0.42
*Calculated composition*					
ME, kcal/kg^4^	3,252	3,255	3,257	3,257	3,257
Crude protein, %	20.65	20.77	20.85	20.81	20.77
Crude fat, %	3.27	3.28	3.29	3.30	3.31
SID Lys:ME^5^	4.31	4.30	4.30	4.30	4.30
Phosphorus, available, %	0.55	0.55	0.55	0.55	0.55
Total Calcium:Phosphorus	1.05	1.05	1.05	1.05	1.05
Zinc, ppm	3,142	3,142	3,142	3,142	3,142
SID Lysine, %	1.40	1.40	1.40	1.40	1.40
Trypsin inhibitor units/mg	0.70	1.60	2.40	3.20	4.00
*SID AA:Lys, %*					
Met + Cys	0.60	0.60	0.60	0.60	0.60
Thr	0.62	0.62	0.62	0.62	0.62
Trp	0.21	0.21	0.21	0.21	0.21
Val	0.65	0.65	0.65	0.65	0.65
Ile	0.58	0.58	0.58	0.58	0.58
Leu	1.03	1.04	1.05	1.05	1.05
His	0.33	0.33	0.34	0.34	0.34
*Analyzed composition*					
Crude protein, %	19.13	19.55	19.93	20.72	20.21
Trypsin inhibitor units/mg	0.25	1.00	1.33	2.69	1.95

^1^Phase 1 fed from d 0 to 9 of the study.

^2^Basemix contained: 25.70% soybean meal, 25.37% whey permeate, 14.63% lactose, 9.0% soy protein concentrate, 3.94% monocalcium phosphate (21%), 2.95% soybean oil, 2.75% HP300, 1.37% salt, 1.33% limestone, 1.67% crystalline amino acids, 0.23% choline chloride, 0.16% trace mineral premix, 0.11% selenium (600 ppm), 0.11% vitamin premix, 0.03% vitamin E, 10.66% commercial pack, and contained 3,333 kcal/kg metabolizable energy, 35.10% lactose, 25.57% crude protein, 4.26% crude fat, 3.88% neutral detergent fiber, 1.57% total calcium, 1.31% total phosphorus, and 279.25 ppm Zn.

^3^Premix provided: 55,010 ppm Zn, 49,015 ppm Fe, 20,0182 ppm Mn, 6,000 ppm Cu, 512 ppm I, 150 ppm Se, 25 ppm Co, 1,501 KIU vitamin A, 543 KIU vitamin D, 12,530 IU vitamin E, 271 mg vitamin K, 11.3 mg vitamin B12, 10.2 mg folic acid, 17,301 mg niacin, 9,503 mg pantothenic acid, and 2,193 mg riboflavin per kilogram of premix.

^4^ME = metabolizable energy.

^5^SID = standardized ileal digestible.

^*^Weighted average of analyzed trypsin inhibitor content across all three phases.

**Table 3.** Diet composition of phase 2, as fed.^1^

Ingredient, %	Dietary Treatment (TIU/mg)^*^				
	0.61	1.22	2.19	3.41	3.51
Corn	56.37	56.92	57.34	57.35	57.36
Soybean meal	28.12	24.10	21.40	21.40	21.40
Soybean flour (20PDI)	-	4.05	5.70	2.85	-
Soybean flour (70PDI)	-	-	1.01	3.86	6.71
Choice white grease	1.00	0.56	0.26	0.25	0.24
Basemix^2^	11.25	11.25	11.25	11.25	11.25
Limestone	0.55	0.55	0.56	0.56	0.56
Monocalcium phosphate	0.41	0.38	0.35	0.35	0.35
Salt	0.75	0.74	0.74	0.74	0.74
Vitamin/Trace mineral premix^3^	0.15	0.15	0.15	0.15	0.15
L-Lysine	0.50	0.47	0.45	0.45	0.45
DL-Methionine	0.25	0.24	0.23	0.23	0.23
L-Threonine	0.20	0.19	0.18	0.18	0.18
L-Tryptophan	0.06	0.06	0.06	0.06	0.06
L-Valine	0.13	0.10	0.09	0.09	0.09
L-Isoleucine	0.04	0.01	-	-	-
Phytase^4^	0.02	0.02	0.02	0.02	0.02
Zinc oxide, 72%	0.21	0.21	0.21	0.21	0.21
*Calculated composition*					
ME^5^, kcal/kg	3,238	3,238	3,238	3,238	3,238
Crude protein, %	20.76	20.93	21.01	20.97	20.93
Crude fat, %	3.68	3.27	3.00	3.00	3.00
SID^6^ Lys:ME	4.23	4.23	4.23	4.23	4.23
Phosphorus, available, %	0.40	0.40	0.40	0.40	0.40
Total Calcium:Phosphorus	1.05	1.05	1.05	1.05	1.05
Zinc, ppm	1,639	1,640	1,640	1,640	1,640
SID Lys, %	1.37	1.37	1.37	1.37	1.37
Trypsin inhibitor units/mg	0.26	1.18	2.10	3.02	3.94
*SID*^6^*AA:Lys, %*					
Met + Cys	0.58	0.58	0.58	0.58	0.58
Thr	0.62	0.62	0.62	0.62	0.62
Trp	0.20	0.20	0.20	0.20	0.20
Val	0.65	0.65	0.65	0.65	0.65
Ile	0.55	0.55	0.55	0.55	0.55
Leu	1.03	1.05	1.06	1.06	1.06
His	0.32	0.33	0.33	0.33	0.33
*Analyzed composition*					
Crude protein, %	20.43	21.70	20.06	19.99	21.21
Trypsin inhibitor units/mg	0.97	1.27	2.47	3.60	4.18

^1^Phase 2 fed from d 10 to 22 of the study.

^2^Basemix contained: 25.70% soybean meal, 25.37% whey permeate, 14.63% lactose, 9.0% soy protein concentrate, 3.94% monocalcium phosphate (21%), 2.95% soybean oil, 2.75% HP300, 1.37% salt, 1.33% limestone, 1.67% crystalline amino acids, 0.23% choline chloride, 0.16% trace mineral premix, 0.11% selenium (600 ppm), 0.11% vitamin premix, 0.03% vitamin E, 10.66% commercial pack, and contained 3,333 kcal/kg metabolizable energy, 35.10% lactose, 25.57% crude protein, 4.26% crude fat, 3.88% neutral detergent fiber, 1.57% total calcium, 1.31% total phosphorus, and 279.25 ppm Zn.

^3^Premix provided: 55,010 ppm Zn, 49,015 ppm Fe, 20,0182 ppm Mn, 6,000 ppm Cu, 512 ppm I, 150 ppm Se, 25 ppm Co, 1,501 KIU vitamin A, 543 KIU vitamin D, 12,530 IU vitamin E, 271 mg vitamin K, 11.3 mg vitamin B12, 10.2 mg folic acid, 17,301 mg niacin, 9,503 mg pantothenic acid, and 2,193 mg riboflavin per kilogram of premix.

^4^Natuphos E (BASF, Ludwigshafen, Germany) provided 2,502,221 FTU/kg available phosphorus.

^5^ME = metabolizable energy.

^6^SID = standardized ileal digestible.

^*^Weighted average of analyzed TIU content across all three phases.

**Table 4.** Diet composition of phase 3, as fed basis.^1^

Ingredient, %	Dietary Treatment (TIU/mg)^*^				
	0.61	1.22	2.19	3.41	3.51
Corn	63.25	63.83	64.20	64.21	64.22
Soybean meal	31.00	26.90	24.27	24.27	24.27
Soybean flour, 20 PDI	-	4.10	5.71	2.85	-
Soybean flour, 70 PDI	-	-	1.02	3.88	6.73
Choice white grease	2.00	1.55	1.27	1.26	1.25
Limestone	0.71	0.72	0.72	0.72	0.72
Monocalcium phosphate	0.81	0.78	0.75	0.75	0.75
Salt	0.74	0.73	0.73	0.73	0.73
Vitamin/Trace mineral premix^2^	0.20	0.20	0.20	0.20	0.20
L-Lysine	0.54	0.51	0.49	0.49	0.49
DL-Methionine	0.29	0.28	0.27	0.27	0.27
L-Threonine	0.22	0.21	0.21	0.21	0.21
L-Tryptophan	0.04	0.05	0.05	0.05	0.05
L-Valine	0.14	0.11	0.09	0.10	0.10
L-Isoleucine	0.03	0.01	-	-	-
Phytase^3^	0.02	0.02	0.02	0.02	0.02
*Calculated composition*					
ME, kcal/kg^4^	3,263	3,263	3,263	3,263	3,263
Crude protein, %	19.77	19.93	20.02	19.98	19.94
Crude fat, %	4.44	4.02	3.76	3.77	3.77
SID^5^ Lys:ME	3.95	3.95	3.95	3.95	3.95
Phosphorus, available, %	0.37	0.37	0.37	0.37	0.37
Total Calcium:Phosphorus	1.05	1.05	1.05	1.05	1.05
SID Lysine, %	1.29	1.29	1.29	1.29	1.29
Trypsin inhibitor units/mg	0.26	1.18	2.10	3.02	3.94
*SID AA:Lysine, %*					
Met + Cys	0.58	0.58	0.58	0.58	0.58
Thr	0.62	0.62	0.62	0.62	0.62
Trp	0.19	0.19	0.19	0.19	0.19
Val	0.65	0.65	0.65	0.65	0.65
Ile	0.54	0.54	0.55	0.55	0.55
Leu	1.06	1.08	1.10	1.09	1.09
Hist	0.32	0.33	0.34	0.34	0.34
*Analyzed composition*					
Crude protein, %	18.97	18.00	18.60	18.48	19.26
Trypsin inhibitor units/mg	0.53	1.28	2.41	3.62	3.78

^1^Phase 3 fed from d 23 to 41 of the study.

^2^Premix provided 55,010 ppm Zn, 49,015 ppm Fe, 20,0182 ppm Mn, 6,000 ppm Cu, 512 ppm I, 150 ppm Se, 25 ppm Co, 1,501 KIU vitamin A, 543 KIU vitamin D, 12,530 IU vitamin E, 271 mg vitamin K, 11.3 mg vitamin B_12_, 10.2 mg folic acid, 17,301 mg niacin, 9,503 mg pantothenic acid, and 2,193 mg riboflavin per kilogram of premix.

^3^Natuphos E (BASF, Ludwigshafen, Germany) provided 2,502,221 FTU/kg available phosphorus.

^4^ME = metabolizable energy.

^5^SID = standardized ileal digestible.

^*^Weighted average of analyzed TIU content across all three phases.

### Animals and Experimental Design

The 41-d study utilized 1,140 newly weaned mixed sex pigs, 18 to 25 d of age (initial body weight [BW] 5.9 ± 0.34 kg; DNA 610 E × DNA 241 F1 and PIC 1050) in a randomized complete block design. Pigs were allotted across 95 pens (12 pigs per pen) into split-sex pens and blocked by initial BW; pens were randomly assigned to one of five dietary treatments (n = 19 replicates per treatment). Pen BW and feed disappearance were collected on d 0, 9, 22, and 41, and average daily gain (ADG), average daily feed intake (ADFI), and feed efficiency (gain-to-feed ratio, G:F) were then calculated by phase. Gain-to-feed, caloric efficiency (ME Mcal/BW gain, Mcal/kg) and lysine efficiency (g SID Lys/BW gain, g/kg) were calculated and plotted against daily TIU/mg intake. Additionally, to evaluate pig BW variability due to dietary treatment, individual pig BW were collected on d 0 and 35 of the study. The change in BW standard deviation (SD) was calculated by subtracting the d 0 BW SD from the d 35 BW SD and reported.

Throughout the 41-d study duration, pigs were allowed ad libitum access to water and feed. Pig medical treatments, removals, and mortalities were monitored and recorded at occurrence by the farm staff in accordance with the standard procedure of the farm and then accounted for in calculations. Fecal consistency scores were recorded daily by the same individual on a 3-point scale, where 1 = normal; 2 = moderately loose; and 3 = severe fecal looseness. These scores were then averaged over each dietary phase to determine the effect of diet on fecal scores.

### Sample Collection

On d 21 of the study, 50 pigs (n = 10 pigs per treatment) were euthanized via captive bolt gun for sample collection. Within each pen, the average BW pig was identified and necropsied. The subset of pens for necropsy was selected based on BW where one light, three medium, and one heavy replicate per sex was selected. Immediately following euthanasia, stomach, distal ileum, and apex colon contents were collected and placed on wet ice or frozen on dry ice. The pH of stomach, ileum and colonic contents was measured using a benchtop meter (Accumet Basic AB315 pH/mV meter, Thermo Fisher Scientific, Waltham, MA).

Segments of the stomach body, mid-jejunum, distal ileum, and apex colon were excised and fixed in 10% neutral buffered formalin for 24 hours, then transferred to 70% ethanol for storage. Fixed sections were then paraffin-embedded into blocks, sectioned, and stained with hematoxylin and eosin at the Iowa State University Veterinary Diagnostic Laboratory (ISUVDL, Ames, IA). An ISUVDL pathologist, blinded to treatment, scored sections of the ileum and colon for lesions on a 4-point scale: 0 = none, 1 = mild, 2 = moderate, and 3 = severe inflammatory lesions or attached bacterial rods. Additionally, ileum and colon morphology was evaluated. Sectioned ileum and colon slides were imaged at 4X magnification using a DP80 Olympus Camera mounted on an OLYMPUS BX 53/43 microscope (Olympus Scientific, Waltham, MA) fitted with a QImaging 12-bit QICAM Fast 1394 camera (Surrey, BC). On each tissue section, approximately 30 to 45 ileal villus height (VH) and crypt depth (CD) pairs and 30 colonic crypts were measured using Image Pro Plus 7.0 (QImaging, Surrey, BC) and averaged per pig.

### Short Chain Fatty Acid and Biogenic Amine Analysis

Short chain fatty acid (SCFA) concentrations in colonic content were analyzed at the Iowa State University W. M. Keck Metabolomics Research Laboratory (Ames, IA) based on methods previously described (Hsu et al., 2019). Briefly, frozen colonic samples were thawed on ice, homogenized and 300 mg of sample were weighed into a microcentrifuge tube. Samples were spiked with 25 µg internal standard (Acetate-d3, Sigma-Aldrich, St. Louis, MO). Then, 1.5 mL of 0.5% phosphoric acid (Thermo Fisher Scientific, Waltham, MA) was added to each sample, homogenized with a Bead Mill 24 Homogenizer (Thermo Fisher Scientific, Inc., Waltham, MA) and then placed in an ice-cold sonicating water bath (Model 2510, Branson Ultrasonics, Brookfield, CT) for 5 min. Samples were vortexed for 2 min and then centrifuged for 7 min at 16,000 g. The supernatant (120 µL) was transferred into a new 1.5 mL microcentrifuge tube, and 480 µL of 0.5% phosphoric acid was added. Short chain fatty acids were extracted using liquid-liquid extraction with 600 µL HPLC-grade 1-butanol (Fisher Scientific, Waltham, MA). Samples were centrifuged for 7 min at 16,000 g to separate and collect the 1-butanol layer for gas chromatography mass spectrometry (GC-MS). Reference SCFA standards (Cayman Chemical, Ann Arbor, MI) were utilized with concentrations ranging from 0.1 to 10,000 µM. Detection of SCFA was done using Agilent 7890C GC and 5975C MS. GC-MS data analysis was conducted using Agilent Mass Hunter Qualitative and Quantitative Analysis (version 10.0). Corresponding SCFA standards were utilized for identification and quantification; additional identification was performed using NIST20 and Wiley 11 GC-MS spectral libraries.

Targeted analysis of biogenic amines and amino acids was performed using frozen colonic content samples submitted to the W.M. Keck Metabolomics Research Laboratory (Iowa State University, Ames, IA). Briefly, free biogenic amines and amino acids were analyzed according to the methods recommended for the EZ:faast kit by GC-MS [Free (Physiological) Amino Acid, Phenomenex, Torrance, CA]. Approximately 100 mg of samples previously frozen at −80 °C were thawed on ice, placed into microcentrifuge tubes, and spiked with 100 µM of internal standards (0.2 mM norvaline, 10% 1-propanol, 20 mM hydrochloric acid). 900 µL of ice cold 75% 1-propanol (HPLC grade 1-propanol 3:1 with LC-MS water, Thermo Fisher Scientific, Waltham, MA) was added, then samples were homogenized with a Bead Mill 24 Homogenizer (Thermo Fisher Scientific, Inc., Waltham, MA) and placed in an ice-cold sonicating water bath (Model 2510, Branson Ultrasonics, Brookfield, CT) for 10 min. Samples were then vortexed and centrifuged at 16,000 g for 10 min. Supernatant was recovered, and 300 µL underwent GC-MS sample prep using methods from EZ:faast kit and was measured against amino acid standards and putrescine and cadaverine standards (Sigma-Aldrich CO., St. Louis, MO). Gas chromatography-mass spectrometry analysis was conducted using Agilent 7890 GC and a model 5975 MS detector (Agilent Technologies, Santa Clara, CA). Identification and quantification was conducted [Automated Mass spectral Deconvolution and Identification System (National Institute of Standards and Technology, Gaithersburg, MD)] and indexed with the GC-MS library, NIST20 and Wiley 11 GC-MS libraries (Agilent Technologies, Santa Clara, CA), and the EZ:faast kit GC-MS libraries (Phenomenex, Torrance, CA). Quantification was determined by the corresponding peak areas relative to the internal standard area and the amine and amino acid standards for each peak. Final quantifications of each were normalized to the mass of the initial colonic samples.

### Statistical Analysis and Calculations

Prior to data analysis, observations ± 3 standard deviations from the overall mean of each parameter were considered outliers and removed from the dataset. Statistical analysis was completed in SAS 9.4 (SAS Inst. Inc., Cary, NC). Normality of all data was verified using the UNIVARIATE procedure. Data were analyzed using PROC MIXED, and the model included the fixed effect of treatment (analyzed TIU/mg) and random effect of block. The GLM procedure was utilized to determine orthogonal polynomial linear and quadratic contrast effects of analyzed TIU concentrations between dietary treatments. Contrast coefficients were determined based on the analyzed TIU within each dietary phase and a weighted average of analyzed TIU across all three dietary phases was determined and utilized for overall data. For all parameters, pen was considered the experimental unit. Differences in least-squares (LS) means were determined using the probability of difference (pdiff) option. Additionally, the Tukey-Kramer adjustment was applied to account for multiple comparisons between dietary treatments. The Fisher’s exact test in the FREQ procedure was utilized for the histological scoring. Data are presented as least-squares (LS) means with a pooled standard error of the mean (SEM) and were considered significant if *P *≤ 0.05 and a tendency for significance if 0.05 ≤ *P *≤ 0.10.

Additionally, daily TIU intake was determined by multiplying daily feed intake by the analyzed TIU/mg concentration of the diet. The relationships between daily TIU intake against G:F, caloric efficiency, and lysine efficiency were determined by calculating the square of the correlation coefficient (R^2^).

## RESULTS

### Growth Performance and Fecal Consistency

Nursery pig growth performance and fecal consistency score by phase are summarized in Table 5. Day 9 BW, ADG, ADFI, and G:F decreased linearly (*P* < 0.001) with increasing dietary active TIU concentrations. Phase 1 BW were reduced in pigs fed diets above 2.19 TIU/mg compared to 0.61 and 1.22 (*P* < 0.001). In phase 2, increasing dietary active TIU concentrations resulted in linear reductions in ADG, ADFI, G:F, and BW (*P* < 0.001). Likewise, a linear decrease in ADG, ADFI, and G:F was observed with increasing dietary active TIU concentrations (*P* < 0.001) in phase 3. Over the entire 41-d study, diets containing on average 3.51 TIU/mg had a 25% reduction in ADG (*P* < 0.001), a 17% reduction in ADFI (*P* < 0.001), and an 8% reduction in G:F (*P* < 0.001) compared to pigs fed the 0.61 TIU/mg control diet. These reductions resulted in a decrease of 4.7 kg and 4.0 kg of final BW in pigs fed 3.41 and 3.51 TIU/mg, respectively, compared to pigs fed the 0.61 TIU/mg diet (*P* < 0.001).

**Table 5.** Growth performance of nursery pigs fed increasing amounts of active trypsin inhibitor protein.

Item	TIU/mg complete feed^1^^,^^2^					SEM	*P* value		
	0.61	1.22	2.19	3.41	3.51		TIU	Linear	Quadratic
*Body weight, kg*									
d 0	5.91	5.91	5.91	5.90	5.91	0.232	0.980	0.986	0.998
d 9	7.35^a^	7.19^ab^	7.06^bc^	6.90^c^	6.98^c^	0.268	<0.001	0.192	0.819
d 22	11.14^a^	10.81^a^	10.27^b^	9.56^c^	9.87^bc^	0.397	<0.001	0.003	0.432
d 41	22.33^a^	21.26^a^	19.80^b^	17.59^c^	18.34^c^	0.686	<0.001	<0.001	0.917
*BW standard deviation*									
d 0	0.33	0.34	0.37	0.31	0.34	0.058	0.598	0.901	0.659
d 35	6.02	5.60	5.87	5.70	5.83	0.258	0.791	0.717	0.668
ΔSD^3^	5.69	5.27	5.50	5.39	5.49	0.267	0.817	0.742	0.612
*Phase 1*									
ADG, kg	0.16^a^	0.14^ab^	0.13^bc^	0.11^c^	0.12^c^	0.006	<0.001	<0.001	0.283
ADFI, kg	0.16^a^	0.15^ab^	0.15^bc^	0.14^c^	0.15^bc^	0.006	<0.001	<0.001	0.819
G:F	0.96^a^	0.92^ab^	0.87^bc^	0.81^c^	0.80^c^	0.020	<0.001	<0.001	0.222
*Phase 2*									
ADG, kg	0.29^a^	0.28^a^	0.25^b^	0.21^c^	0.22^bc^	0.012	<0.001	<0.001	0.215
ADFI, kg	0.42^a^	0.42^a^	0.38^b^	0.35^c^	0.36^bc^	0.015	<0.001	<0.001	0.413
G:F	0.68^a^	0.67^a^	0.64^ab^	0.58^c^	0.62^bc^	0.011	<0.001	<0.001	0.182
*Phase 3*									
ADG, kg	0.59^a^	0.55^b^	0.50^c^	0.43^d^	0.45^d^	0.018	<0.001	<0.001	0.857
ADFI, kg	0.80^a^	0.76^ab^	0.70^bc^	0.61^d^	0.66^cd^	0.027	<0.001	<0.001	0.796
G:F	0.74^a^	0.73^ab^	0.72^abc^	0.70^bc^	0.68^c^	0.010	<0.001	<0.001	0.626
*Overall*									
ADG, kg	0.40^a^	0.37^b^	0.34^c^	0.28^d^	0.30^d^	0.012	<0.001	<0.001	0.707
ADFI, kg	0.54^a^	0.51^a^	0.47^b^	0.42^c^	0.45^bc^	0.017	<0.001	<0.001	0.665
G:F	0.74^a^	0.73^ab^	0.71^b^	0.68^c^	0.68^c^	0.007	<0.001	<0.001	0.624
*Fecal Scores* ^4^									
Phase 1	1.61	1.59	1.57	1.60	1.65	0.030	0.417	0.403	0.193
Phase 2	1.98	1.92	1.92	1.92	1.95	0.037	0.515	0.616	0.268
Phase 3	1.80	1.85	1.83	1.81	1.85	0.028	0.491	0.688	0.491
Overall	1.81	1.81	1.80	1.80	1.83	0.020	0.553	0.802	0.551
Removals, %^5^	1.75	1.75	2.19	2.63	2.63	0.950	0.915	0.360	0.929

^a,b,c,d^Means with differing superscripts differ significantly (*P < *0.05).

^1^TIU = trypsin inhibitor units.

^2^n = 19 replicates per treatment.

^3^SD = standard deviation (individual pig BW collected on d 0 and 35 of study, represents the difference between d 35 SD and d 0 SD).

^4^Pen average fecal scores (1 to 3 scale): 1 = normal semi-solid feces, 2 = soft, and 3 = severe looseness.

^5^Accounts for combined mortalities and removals.

The change in standard deviation (ΔSD) of d 35 and d 0 BW, mortality, and removals did not differ between dietary treatment (*P* > 0.10; Table 5). Fecal consistency was unaffected by dietary active TIU concentrations during phases 1, 2, 3, or overall (*P* > 0.10; Table 5).

**Figure 1.**
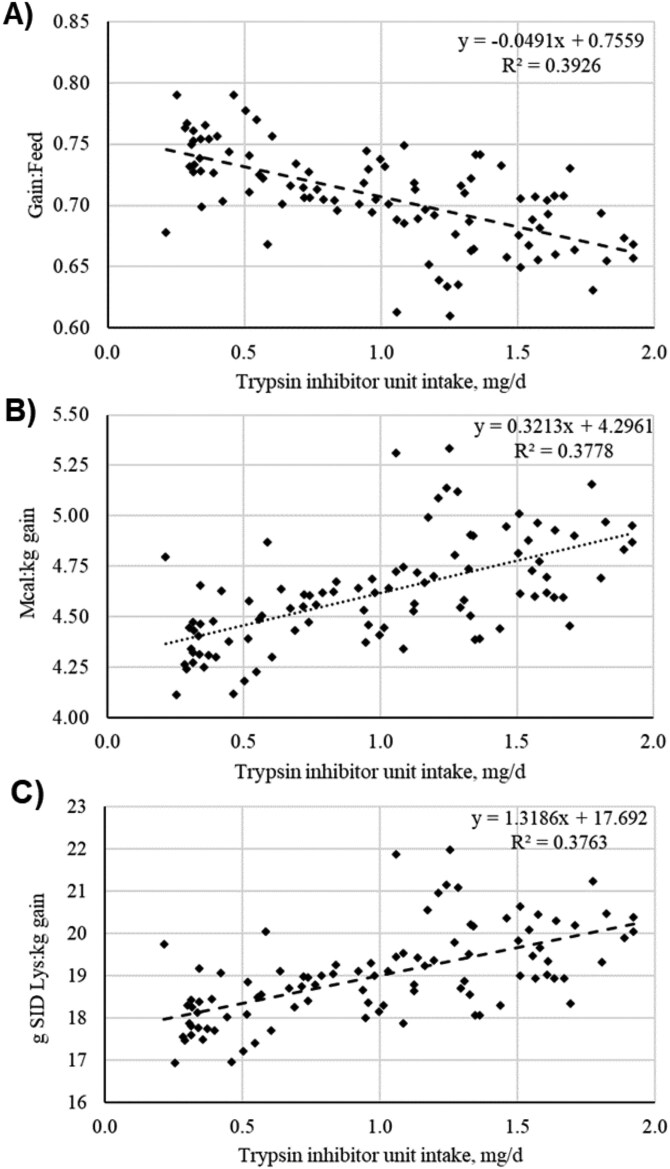
The relationship between soybean active trypsin inhibitor unit intake (mg/d) per day and overall (0 to 41 d) nursery pig (**A**) Feed efficiency (gain:feed); (**B**) Caloric efficiency (Mcal of ME:kg BW gain); and (**C**) Lysine efficiency (g of SID Lys:kg BW gain).

Correlation coefficients between TIU intake (mg/d) and efficiency parameters were examined. There were moderate negative relationships between TIU intake and G:F (R^2^ = 0.393; Figure 1A), caloric efficiency (R^2^ = 0.378; Figure 1B), and lysine efficiency (R^2^ = 0.376; Figure 1C). Together, these data show that feed efficiency and both energy and lysine efficiency for growth decreased as daily TIU intakes increased.

### Intestinal Contents pH, Short Chain Fatty Acid and Targeted Bioamine Concentrations

Digesta pH of the stomach, ileal, and colonic contents were measured after 21 d on test diets (Table 6). Stomach and ileal digesta pH on d 21 were not different between dietary treatments (*P* > 0.10). However, colonic digesta tended to have a higher pH in pigs fed the 3.51 TIU/mg diet compared to the other treatment groups (*P* = 0.094).

**Table 6.** Intestinal pH, morphology, short chain fatty acid (SCFA) and biogenic amine concentrations in nursery pigs following 21 d of soybean active trypsin inhibitor protein consumption.

Item	TIU/mg complete feed^1^^,^^2^						*P* value		
	0.61	1.22	2.19	3.41	3.51	SEM	TIU	Linear	Quadratic
*pH*									
Stomach	3.39	3.00	3.32	2.60	2.91	0.365	0.538	0.193	0.752
Ileum	6.07	5.45	6.06	5.99	6.19	0.231	0.200	0.284	0.397
Colon	5.52^y^	5.55^y^	5.50^y^	5.49^y^	5.74^x^	0.071	0.094	0.265	0.365
*Intestinal Morphology*									
Ileum villus height, µm	361	355	362	330	315	20.3	0.284	0.078	0.380
Ileum crypt depth, µm	277^a^	260^ab^	247^ab^	226^b^	240^ab^	12.4	0.026	0.004	0.583
Ileum VH:CD	1.38	1.42	1.54	1.52	1.39	0.098	0.428	0.574	0.306
Colon crypt depth, µm	477	471	456	438	479	15.3	0.244	0.347	0.471
*Colonic SCFA,* µmoles/g									
Acetate	71.0	79.9	73.5	74.0	68.9	4.30	0.372	0.490	0.318
Butyrate	16.1	18.3	16.1	16.5	13.8	1.67	0.367	0.288	0.469
Isobutyrate	1.2	1.1	1.2	1.3	1.4	0.18	0.806	0.397	0.444
Isovalerate	0.8	0.6	0.8	0.8	0.9	0.16	0.838	0.528	0.523
Propionate	30.0	34.6	32.9	31.2	29.5	2.22	0.464	0.533	0.135
Valerate	3.2	3.2	2.7	3.4	2.9	0.50	0.822	0.856	0.575
Total SCFA	122.3	137.8	127.1	127.2	117.4	6.96	0.266	0.399	0.238
*Amines,* µmoles/g									
Alanine	10.9	8.7	10.4	10.4	9.9	0.98	0.503	0.919	0.873
Glycine	4.9	3.9	4.7	4.7	4.7	0.57	0.703	0.778	0.636
Valine	4.1	3.0	3.8	3.6	3.8	0.50	0.602	0.867	0.547
Leucine	4.0	3.0	3.8	3.7	3.8	0.48	0.664	0.903	0.565
Proline	4.2	3.3	4.1	3.9	3.9	0.53	0.738	0.970	0.962
Aspartic acid	4.0	4.3	4.2	4.3	3.8	0.59	0.938	0.829	0.780
Glutamic acid	16.6	15.8	15.9	17.1	16.4	2.14	0.986	0.965	0.707
Ornithine	6.0	4.0	4.7	3.3	5.2	0.97	0.255	0.568	0.341
Lysine	24.7	18.1	24.7	19.8	21.6	3.86	0.362	0.829	0.232
Cadaverine	18.1	14.3	18.3	22.5	17.8	7.48	0.947	0596	0.847
Putrescine	3.5	2.6	2.8	2.6	2.9	0.53	0.668	0.555	0.656
Total Amines	117.7	93.8	112.6	111.3	109.2	12.30	0.653	0.981	0.559

^a,b^Means with differing superscripts differ significantly (*P < *0.05).

^x,y^Means within a row tend to differ at 0.05 ≤ *P *≤ 0.10.

^1^n = 10 pigs/treatment.

^2^TIU = trypsin inhibitor units.

Colonic SCFA and biogenic amine concentrations are reported in Table 6 for pigs fed dietary treatments for 21 d. Dietary active TIU concentrations did not influence colonic individual or total SCFA concentrations (*P* > 0.10). No differences in dietary treatment were reported for cadaverine and putrescine colonic concentrations (*P *> 0.10). Furthermore, no significant dietary differences were observed for other measured amines or total amine concentrations (*P* > 0.10).

### Intestinal Histopathology and Morphology

Sections of the stomach, ileum, and colon were evaluated for gastritis, villus atrophy, and colitis, respectively (Table 7). Additionally, ileal VH, CD, VH:CD, and colonic CD morphology were assessed (Table 6). There was no evidence that dietary active TIU concentrations influenced the incidence of gastritis, villus atrophy, or colitis in these healthy nursery pigs (*P* > 0.10). Ileal VH tended to decrease as TIU concentrations increased (*P* = 0.078), while ileal CD significantly decreased (*P* = 0.004); however, VH:CD was unaffected (*P* > 0.10). Colon CD was not influenced by dietary treatment (*P* > 0.10).

**Table 7:** Intestinal histopathology assessment of nursery pigs fed increasing concentrations of soybean active trypsin inhibitor protein for 21 d post-weaning.

Item	TIU/mg complete feed^1^^,^^2^					*P* value
	0.61	1.22	2.19	3.41	3.51	TIU
*Gastritis* ^3^						
0	5/10	5/10	6/10	2/10	2/10	0.239
1	3/10	3/10	3/10	6/10	2/10	
2	2/10	2/10	1/10	2/10	6/10	
3	0/10	0/10	0/10	0/10	0/10	
*Atrophy* ^3^						
0	6/10	7/10	9/10	6/10	7/10	0.603
1	4/10	3/10	1/10	4/10	3/10	
2	0/10	0/10	0/10	0/10	0/10	
3	0/10	0/10	0/10	0/10	0/10	
*Colitis* ^3^						
0	5/10	3/10	4/10	5/10	7/10	0.503
1	4/10	7/10	6/10	4/10	3/10	
2	1/10	0/10	0/10	1/10	1/10	
3	0/10	0/10	0/10	0/10	0/10	

^1^TIU = trypsin inhibitor units.

^2^n = 10 replicates per treatment.

^3^Measured on a 0 to 3 scale: 0 = none, 1 = mild, 2 = moderate, and 3 = severe lesions.

## DISCUSSION

Soybeans contain significant amounts of trypsin inhibitor proteins, which inhibit the digestive activity of the protease trypsin, impeding protein digestion and ultimately reducing growth and feed efficiency of monogastric species (Liener, 1976). The mechanism by which trypsin inhibitors impair growth involves negative feedback that augments pancreatic secretions, which is stimulated by an increased release of cholecystokinin as a result of insufficient protein digestion in the intestinal lumen (Liener, 1994; Lajolo and Genovese, 2002). As a result, both pancreatic hypertrophy and hyperplasia are often reported in some animals, such as broiler chickens (Clarke and Wiseman, 2005), resulting in hypersecretion of pancreatic digestive enzymes (Schneeman et al., 1977). Younger animals may be more prone to the negative effects of trypsin inhibitor proteins in complete feed exceeding 3 TIU/mg (Nisley et al., 2024). Furthermore, nursery pigs commonly exhibit an increased incidence of diarrhea, which has been associated with feeding high crude protein diets and increased protein fermentation and production of amines (Zhang et al., 2020). Therefore, our objective was to further validate these findings by assessing the impact of increasing soybean protein-derived active TIU concentrations on nursery pig performance, body weight variability, and general health in nursery-aged pigs.

A recent survey of Midwest United States soybean meals reported that ranges of 2 to 20 TIU/mg can be found (Kim and Krishnan, 2023). Thus, for nursery pig practical diet formulations containing 15% to 30% soybean meal inclusion rates, complete feed TIU/mg could range from 0.3 to 6 TIU/mg. The targeted dietary TIU concentrations in the current study were chosen to model the range to which soybean meals of varying TIU concentrations are typically incorporated into complete diets. Although it is accepted that the heating of soybean meal deactivates a significant proportion of trypsin inhibitor proteins, processing methods differ in their effectiveness. Deactivation and reductions of trypsin inhibitor proteins can range from 55% (Carvalho et al., 2013) to 96% (Osman et al., 2002). Consequently, soy products may still contain trypsin inhibitors in either their active or deactivated form. To account for this variability, diets in this study were formulated based on active trypsin inhibitor protein activity while maintaining equivalent total protein levels across treatments.

Soybean meal can vary in composition, quality and TIU content depending on the processing procedures (Gaffield et al., 2024). Due to this variability, researchers have typically formulated diets containing soybean meal and a blend of raw ground soybeans to achieve varying complete feed TIU levels (Royer et al., 2022; Miller et al., 2024; Nisley et al., 2024). To achieve high TIU diets, low-fat soybean flour was utilized to provide high levels of TIU protein instead of full-fat ground soybeans. Compared to soybean meal, raw soybeans are higher in TIU and crude fat, but lower in crude protein, amino acids, and minerals (NRC, 2012). Although low, relative to soybean meal low-fat soybean flour contained slightly higher crude fat and crude protein, lower ADF and NDF, while containing similar indispensable and dispensable amino acid profiles (Table 1). Further, soybean meal is typically processed and heat treated longer that soybean flour, resulting in lower TIU. The differences in heat treatment by soybean processing plants results in large variation in soybean meal TIU (Gaffield et al., 2024). Soybean hulls, waxes and gums may also be added back to soybean meal, contributing to variability in fiber and general composition (Gaffield et al., 2024). Therefore, with less confounding effects of ingredient fat and fiber, soybean flour may be a better ingredient than whole beans due to model soybean meal and TIU protein. The soybean meal sourced for this current study contained a low TIU level. This level was lower than what has been reported in survey reports of U.S. soybean meal (Kim and Krishnan 2023; Gaffield et al., 2024). The low soybean meal TIU could be a result of over-processing and excessive heat or pressure during toasting, drying, or extrusion. While heat is essential to deactivate antinutritional factors, such as TIU, excessive heat damages proteins and reduces their nutritional value to pigs (González-Vega et al., 2011). However, based on the feed intake and growth rates of the nursery pigs fed the lower TIU diets containing high soybean meal, minimal evidence would suggest that amino acid digestibility and bioavailability were impacted due to over-processed soybean meal.

It is well documented that high dietary trypsin inhibitor protein concentrations compromise growth in monogastric species (Woyengo et al., 2017). This reduction in growth is attributed to decreased protein digestibility and amino acid bioavailability, which impairs weight gain, as observed in rats (Kakade et al., 1973; Schneeman et al., 1977; Gu et al., 2010), chickens (Palacios et al., 2004; Clarke and Wiseman, 2005), and pigs (Yen et al., 1974). While few studies have directly examined the level at which trypsin inhibitor proteins impact pig performance (Royer et al., 2022), extensive research has explored the effects of whole soybean and soybean meal heat treatments (Combs et al., 1967; Herkelman et al., 1992) and soybean cultivars (Cook et al., 1988; Palacios et al., 2004) on pig performance. In 30 kg growing pigs, Royer et al. (2022) reported attenuated feed intake, weight gain, and feed efficiency as dietary TIU concentrations exceeded 2 TIU/mg. Similarly, in 40 kg BW grower pigs, Miller et al. (2024) reported reductions in BW gain of 15% and 22.4% and feed efficiency of 14.5% and 25% in diets containing 6.49 and 9.38 TIU/mg, respectively, compared to 0.99 TIU/mg, while feed intake was unaffected. However, in nursery pigs, the effects of dietary TIU concentrations appear to be more severe. Nisley et al. (2024) observed linear reductions in ADG and G:F, and a tendency for decreased ADFI, in weaned pigs fed diets ranging from 0.38 to 5.79 TIU/mg over 42 d. These resulted in an approximately 10 kg reduction in BW in pigs fed 5.79 TIU/mg compared to those fed 0.38 TIU/mg (Nisley et al., 2024). In agreement, the current study reported that weight gain and feed efficiency began to decline at concentrations above 1.22 TIU/mg, with the most pronounced reductions occurring at 3.41 and 3.51 TIU/mg, where BW losses of 4.7 kg and 4.0 kg, respectively, were observed. Additionally, linear reductions in feed intake were observed in the current study, all of which are consistent with Nisley et al. (2024), who concluded that the most severe impacts to growth, feed intake, and efficiency were in pigs fed diets exceeding 3.16 TIU/mg. Herein, performance is impaired at levels exceeding 1.22 TIU/mg, which may be driven by the high amount of treatment replicates compared to Nisley et al. (2024) (19 pens/treatment vs. 10 pigs/treatment, respectively).

Lysine and caloric efficiency were calculated and regressed against daily TIU intake in the present study to determine the effect of TIU on nutrient utilization. Trypsin inhibitors can impact free amino acids in addition to peptides (Liener et al., 1949); therefore, it is reasonable to expect lysine efficiency to worsen with higher amounts of TIU in the diet, as was demonstrated in the current study. Likewise, caloric efficiency worsened as TIU concentrations increased, which may also be attributed to decreased protein digestion. As protein sources such as soybean meal are relatively high energy-containing ingredients and can contribute 15 to 25% of the energy concentration of a nursery pig diet (NRC, 2012), a substantial amount of energy may be unavailable to the animal due to high levels of trypsin inhibitor protein in the diet.

Trypsin inhibitors reduce nitrogen and protein digestibility (Goebel and Stein, 2011), resulting in increased amino acids and peptides reaching the hindgut (Liener, 1976). Similarly, nursery pigs fed high crude protein diets may experience substantial amounts of undigested protein entering the large intestine (Stein et al., 2007; Heo et al., 2009). This creates an opportunity for protein fermentation, a process associated with increased diarrhea incidence (Wellock et al., 2007; Zhang et al., 2020). The proposed mechanism linking proteolytic fermentation to diarrhea in young pigs involves reduced sodium and water absorption in the colon due to elevated H_2_S levels (Roediger et al., 1997). However, the relationship between high crude protein diets and diarrhea incidence in nursery pigs has been inconsistent. While some studies report increased diarrhea with higher dietary crude protein content (Heo et al., 2009; Batson et al., 2021; Limbach et al., 2021), others find no effect on fecal consistency (Nyachoti et al., 2006; Htoo et al., 2007). To our knowledge, limited research exists on the association between dietary active trypsin inhibitor concentrations and fecal consistency. Consistent with the current findings, Nisley et al. (2024) reported no changes in fecal consistency in nursery pigs fed diets with active TIU levels ranging from 0.22 to 5.79 TIU/mg over the first 2 wk. Similarly, in growing pigs, Royer et al. (2015) reported no change in fecal consistency scores over the duration of the study. The lack of observed differences between high crude protein diets and those that have reduced nitrogen digestibility (i.e., high in trypsin inhibitor protein concentrations) may be attributed to the formulation of diets to a constant crude protein level, rather than the undigested protein reaching the hindgut from trypsin inhibition.

Short-chain fatty acids and biogenic amines are end products of amino acid catabolism and microbial proteolytic fermentation (Zhang et al., 2020) and serve as proxies for microbial activity on the undigested protein that reaches the hindgut. To our knowledge, the effect of TIU on hindgut fermentation is limited. It is generally hypothesized that increasing crude protein levels elevate SCFA (Nisley, 2023) and amine concentrations in the lumen and digesta of pigs, but often do not alter pH (Zhang et al., 2020). However, studies testing this hypothesis have produced inconsistent results regarding metabolite production and digesta pH. Nyachoti et al. (2006) observed linear decreases in ileal SCFA concentrations in early-weaned pigs when dietary crude protein was reduced from 23% to 17%. Additionally, these authors indicated no impact of crude protein level on duodenum or jejunum digesta pH, but a quadratic effect on ileal digesta pH, where 23% and 17% crude protein diets had higher values than diets with 21% and 19% protein (Nyachoti et al., 2006). Similarly, Heo et al. (2009) did not report pH measurements but did report lower SCFA concentrations in nursery pigs fed a low-protein diet (18%) compared to a high-protein diet (24%) after 7 d, although these differences did not persist throughout the 14-d period. In contrast, Htoo et al. (2007) found no differences in SCFA or biogenic amine concentrations, or ileal digesta pH, in early-weaned pigs fed low (20%) and high (24%) crude protein diets. Additionally, Limbach et al. (2021) observed decreases in ileal digesta pH as crude protein in the diet increased from 16% to 22%, but no change in SCFA concentrations. In the current study, all diets were formulated to a similar crude protein level (21%). Despite this, no significant differences in SCFA, biogenic amine concentrations, or digesta pH were observed, which corresponded with no differences in fecal consistency. These findings suggest that dietary crude protein level drives differences in hindgut protein fermentation rather than undigestible protein (i.e., trypsin inhibitor proteins).

Intestinal morphology is influenced by dietary crude protein levels, undigested protein reaching the large intestine (Zhang et al., 2020), and protein sources (Vente-Spreeuwenberg et al., 2004). However, the knowledge of the impact of active trypsin inhibitor proteins on intestinal morphology is limited. Studies examining high crude protein diets have reported varying effects on intestinal morphology. Limbach et al. (2021) observed a decrease in ileal CD when dietary crude protein levels were reduced from 22% to 16%, although VH and the VH:CD ratio were unaffected. Similarly, Chen et al. (2018) noted improved VH in pigs fed an 18% crude protein diet compared to a 12% and 15% crude protein diet, while Nyachoti et al. (2006) reported no changes in ileal VH or CD with a reduction in crude protein from 23% to 17%. In the current study, VH tended to decrease and CD linearly decreased as dietary active TIU concentrations increased. Given the close association between intestinal morphology and nutrient absorption (Pluske et al., 2018), it is reasonable to infer that reduced nutrient digestibility impairs digestive capacity. In the colon, deeper crypts are often associated with higher dietary protein levels (Chen et al., 2018) and diarrhea in pigs (Zhang et al., 2020). However, colonic CD did not differ among diets with increasing TIU concentrations in this study. This lack of differences may be attributed to the similar crude protein levels across diets and the absence of fecal consistency changes.

Soybean protein sources are commonly used in nursery pig diets to supply amino acids and energy. However, understanding the potential negative implications of incorporating ingredients that may contain high amounts of antinutritional factors, such as trypsin inhibitor proteins, is critical. In conclusion, the findings from the current study indicate that nursery pig performance is attenuated as dietary active trypsin inhibitor concentrations from soybean protein increase. Importantly, minimal adverse effects on intestinal health metrics were observed. The results demonstrate that performance losses occur at active TIU concentrations above 1.22 TIU/mg in complete feed in nursery pigs, which is lower than initially hypothesized.
